# Supplementary material for: The internal and external factors influencing talent development of athletic talent among Saudi Arabia’s twice exceptional elite athletes: A comprehensive study
Source: PLoS One. 2024 Dec 27;19(12):e0304049. doi: 10.1371/journal.pone.0304049 (PMC11676560; doi:10.1371/journal.pone.0304049)
Supplement: Attachment — Submitted filename: Response to Reviewers.docx [file pone.0304049.s001.docx]

Dear Dr. Tien-Chin Tan.

I would like to extend my sincere gratitude to you for your time and effort in reviewing our manuscript. I deeply appreciate the meticulous attention you have given to the manuscript and the valuable feedback you have provided.

Your insights and suggestions have been instrumental in identifying areas for improvement. The clarity and depth of your comments have significantly contributed to enhancing the quality of the manuscript. The authors are grateful for your dedication to ensuring the highest standards of academic rigor and for your constructive assistance in refining the work.

Thank you once again for your invaluable support and for helping us improve this research.

Respond to all comments received via email

| No. | Comments | Response |
| --- | --- | --- |
| Journal requirements: | | |
| contact information for a Data Ethics Committee, | | 00966135899773 [ialjreesh@kfu.edu.sa](mailto:ialjreesh@kfu.edu.sa) |
| 1 | When submitting your revision, we need you to address these additional requirements | Done |
| 2 | We note that the grant information you provided in the ‘Funding Information’ and ‘Financial Disclosure’ sections do not match. | Done |
| 3 | Thank you for stating the following in the Acknowledgments Section of your manuscript: "In The authors extend their appreciation to the King Salman center For Disability Research for funding this work through Research Group no KSRG-2022-090." We note that you have provided funding information that is not currently declared in your Funding Statement. However, funding information should not appear in the Acknowledgments section or other areas of your manuscript. We will only publish funding information present in the Funding Statement section of the online submission form. Please remove any funding-related text from the manuscript and let us know how you would like to update your Funding Statement. Currently, your Funding Statement reads as follows: “The authors received no specific funding for this work.” Please include your amended statements within your cover letter; we will change the online submission form on your behalf. | This work is supported by the King Salman Center for Facebook Research Group No. KSRG-2022-090 |
| 4 | We note that your Data Availability Statement is currently as follows: "All relevant data is within the manuscript and it's supporting information files."  Please confirm at this time whether your submission contains all raw data required to replicate the results of your study. Authors must share the “minimal data set” for their submission. PLOS defines the minimal data set to consist of the data required to replicate all study findings reported in the article, as well as related metadata and methods (https://journals.plos.org/plosone/s/data-availability#loc-minimal-data-set-definition).  For example, authors should submit the following data:  - The values behind the means, standard deviations and other measures reported;  - The values used to build graphs;  - The points extracted from images for analysis.  Authors do not need to submit their entire data set if only a portion of the data was used in the reported study.  If your submission does not contain these data, please either upload them as Supporting Information files or deposit them to a stable, public repository and provide us with the relevant URLs, DOIs, or accession numbers. For a list of recommended repositories, please see https://journals.plos.org/plosone/s/recommended-repositories.  If there are ethical or legal restrictions on sharing a de-identified data set, please explain them in detail (e.g., data contain potentially sensitive information, data are owned by a third-party organization, etc.) and who has imposed them (e.g., an ethics committee). Please also provide contact information for a data access committee, ethics committee, or other institutional body to which data requests may be sent. If data are owned by a third party, please indicate how others may request data access. | To protect the rights of the participants, certain restrictions were enforced, and the referees were provided with an ethical approval letter for the research. This approval justifies the decision not to publish the transcription of the interview data, particularly because the participants are individuals with disabilities. They were informed during the interviews and the review of achievement files that their data would remain confidential and would not be publicly disclosed. Additionally, the researchers included Appendix No. 1, which outlines the minimum essential data required.  Regarding participant data, the researchers adhere to the ethical approval framework issued by the Deanship of Scientific Research at King Faisal University, Al-Ahsa, under reference number KFU-REC-2022-OCT-ETHICS195, dated October 11, 2022. This framework ensures the protection of participants' rights in research activities and projects, and the confidentiality of their data, as stated in the following text:  "The researchers are responsible for storing, safeguarding, and securing the participants' data in research projects."  In lieu of this, all necessary data has been provided to the esteemed reviewers to enable them to access the essential information required to replicate the results and verify the accuracy of participant data and qualitative analysis, as detailed in Table (1) Analysis of Participant Data.   |
| 5 | When completing the data availability statement of the submission form, you indicated that you will make your data available on acceptance. We strongly recommend all authors decide on a data sharing plan before acceptance, as the process can be lengthy and hold up publication timelines. Please note that, though access restrictions are acceptable now, your entire data will need to be made freely accessible if your manuscript is accepted for publication. This policy applies to all data except where public deposition would breach compliance with the protocol approved by your research ethics board. If you are unable to adhere to our open data policy, please kindly revise your statement to explain your reasoning and we will seek the editor's input on an exemption. Please be assured that, once you have provided your new statement, the assessment of your exemption will not hold up the peer review process. | Link of data statistics, Analysis of Participant Athletes' Data, Comparative Analysis and Sequence of Interview Sessions, Their Duration, and Word Count file  <https://docs.google.com/spreadsheets/d/1OTM8hsb8vPJquYCoke2OtDIwLi-gX36F/edit?usp=drive_link&ouid=107741125684008076542&rtpof=true&sd=true> |
| Comments to the Author | | |
| 1 | Is the manuscript technically sound, and do the data support the conclusions?  The manuscript must describe a technically sound piece of scientific research with data that supports the conclusions. Experiments must have been conducted rigorously, with appropriate controls, replication, and sample sizes. The conclusions must be drawn appropriately based on the data presented.  Reviewer #1: Partly  Reviewer #2: Partly | Table "1" provides the minimum essential data for the reviewers to match the interview data with the analysis results. |
| 2 | Has the statistical analysis been performed appropriately and rigorously?  Reviewer #1: N/A  Reviewer #2: N/A | This study is purely qualitative, which aligns with the approach used in most talent development research. The primary objective of this methodology is to investigate the lives of exceptional individuals in a specific field to identify the factors contributing to their success. For example, studies by Bloom & Sosniak (1981) and Dai & Li (2020) employed a similar methodology. Additionally, the study by Côté et al. (2005) utilized interviews to collect primary data from athletes, a method also employed in this research while ensuring the confidentiality of participant data. |
| 3 | Have the authors made all data underlying the findings in their manuscript fully available?  The PLOS Data policy requires authors to make all data underlying the findings described in their manuscript fully available without restriction, with rare exception (please refer to the Data Availability Statement in the manuscript PDF file). The data  should be provided as part of the manuscript or its supporting information, or deposited to a public repository. For example, in addition to summary statistics, the data points behind means, medians and variance measures should be available. If there  are restrictions on publicly sharing data—e.g. participant privacy or use of data from a third party—those must be specified.  Reviewer #1: Yes  Reviewer #2: Yes | The study has implemented measures to protect participants' rights and has provided the esteemed reviewers with an ethical approval letter for the research, which justifies the decision not to publish the interview transcripts. This is due to the participants being individuals with disabilities, and they were informed during the interviews and the review of achievement records about the confidentiality of the data used and the restriction on its public dissemination. Additionally, the researchers have provided Table 1, which outlines the minimum data required to enable the reviewers to cross-check the interview data with the analysis results. |
| 4 | Is the manuscript presented in an intelligible fashion and written in standard English?  PLOS ONE does not copyedit accepted manuscripts, so the language in submitted articles must be clear, correct, and unambiguous. Any typographical or grammatical errors should be corrected at revision, so please note any specific errors here.  Reviewer #1: No  Reviewer #2: Yes | All instructions and forms in the scientific journal PLOS ONE were followed. This was also taken into consideration when responding to the referees in the manuscript submitted after reviewing the reviewers. |
| Review Comments to the Author | | |
| Please use the space provided to explain your answers to the questions above. You may also include additional comments for the author, including concerns about dual publication, research ethics, or publication ethics. (Please upload your review as an attachment if it exceeds 20,000 characters) | | |
| Reviewer #1 | It is academically and practically beneficial to explore the developmental elements of internal and external influences on this group of twice-exceptional elite athletes. However, there are still several significant issues to consider and clarify.  The title, purpose, and content of the article should be consistent, relevant literature should be augmented, the framework for the analysis should be clarified, the interviewees and data collection should be clarified, and the findings and discussion  should be reconsidered. These issues have resulted in the current study's conclusions being only partially supported. Additionally, the entire article has a number of formal problems that need to be addressed. These issues and problems have had a  significant impact on the overall quality of the current study. Please consider the following suggestions before submitting the article to relevant journals. | We would like to clarify the following points in response to your comments:  Consistency between Title, Objective, and Content: We recognize the importance of consistency between the title, objective, and content of the article. Based on your feedback, we have reviewed and adjusted the title and objective to better align with the research content. The title has been revised to accurately reflect the scope and specific aims of the study.  Enhancement of Relevant Literature: We have expanded and improved the review of relevant literature to include additional studies that highlight previous research and current developments in this field. This enhancement strengthens the theoretical framework and adds depth to our analysis.  Clarification of Analytical Framework: We have clarified the analytical framework used in the study, including the methods employed and the criteria we applied for data analysis. This aims to provide a clearer understanding of the research methodology.  Clarification of Participants and Data Collection: Additional details regarding the participants in the interviews and the data collection procedures have been provided. We have outlined the criteria for selecting participants and the methods of data collection to ensure transparency in the research process.  Reevaluation of Results and Discussion: We have reexamined the results and discussion based on the provided feedback. The discussion has been updated to include a more in-depth analysis of the results and their implications for the study.  Addressing Formal Issues: The entire manuscript has been reviewed to address the formal issues mentioned. Errors have been corrected, and the manuscript has been ensured to meet the established academic standards. |
| Introduction | | |
| 1 | In the title of this paper, the authors state that they investigate the factors influencing the development of talent and performance among elite twice-exceptional athletes. This is inconsistent with the research objective, “This study investigates the internal and external factors influencing the development of athletic talent among elite twice-exceptional athletes” as stated in lines 85-86. Moreover, the authors did not present an analysis of the factors influencing elite twice-exceptional athletes' performance. Therefore, the authors need to pay more attention to the study title, purpose, and content consistency. | The terminology has been standardized, and the term "performance" has been removed, with a focus solely on identifying the internal and external factors according to the study’s objectives.  Title Modified:" Internal and External Factors Influencing the Development of Athletic Talent among Twice-Exceptional Athletes in Saudi Arabia: A Comprehensive Study" |
| 2 | The authors’ review of existing literature on the factors influencing the development of athletes in competitive sports, or on the factors affecting the development of elite twice-exceptional athletes, is not sufficient and comprehensive. The current  authors’ review of relevant previous studies discusses factors at the individual and environmental levels. However, previous studies such as De Bosscher et al. (2006) mentioned that the factors affecting athletic talents development and performance  can be categorized into micro, meso, and macro levels. Therefore, the authors may need to refer to De Bosscher et al. (2006) and related previous studies to reorganize the review of related studies.  Veerle De Bosscher, Paul De Knop, Maarten Van Bottenburg & Simon Shibli (2006). A Conceptual Framework for Analysing Sports Policy Factors Leading to International Sporting Success, European Sport Management Quarterly, 6:2, 185- 215, DOI:  10.1080/16184740600955087 | The reviewers emphasized the importance of enriching the study by reviewing the work of De Bosscher et al. (2006), which identified three levels for classifying factors leading to international sports success, such as an integrated national policy. This study presents an "ideal model for achieving excellence in the care of elite athletes." However, our current study differs in that it addresses the unique needs of individuals with disabilities, who differ significantly from the ideal care model provided to non-disabled individuals. Therefore, our research focuses on examining the success pathways of participants, identifying factors directly related to the specific needs of these cases, and analyzing care levels. We aim to find points of intersection for success through the mega model and identify internal and external factors.  Additionally, upon reviewing the studies and literature references used in this research, it is evident that many elements highlighted in the De Bosscher et al. (2006) model are reflected, to varying degrees, in the results and discussion of our study. However, these are presented individually and less generally than in the aforementioned study. This distinction highlights the unique focus of research on individuals with disabilities, emphasizing participant specificity and uniqueness. |
| Materials and Methods | | |
| 1 | The authors’ description of the respondents in Table 1 allows readers to understand the respondents' composition. However, this section should generally include the respondent's code number, interview date, and their characteristics, such as disabilities classification, specialty sports items, and sports performance. By doing so, readers will be able to better comprehend the source of evidence. This in turn should provide a more convincing picture of the findings. | All requirements were added in Table (1) |
| 2 | 2. In lines 194-196, the authors state that “the integration of peer review and synthesis further strengthened the analytical process, enhancing the credibility and impact of the research outcomes suitable for publication in high-impact international scientific journals”. However, it seems arbitrary for the authors to assert that their research outcomes are suitable for publication in high-impact scientific journals. Researchers should focus on explaining how their research has enhanced data credibility. | The inappropriate phrase was removed from the revised version in lines 194-196, and the line order was adjusted accordingly.  The following passage has been added to clarify the credibility of the interviews and qualitative data analysis:  Data from the participants were collected using the study tool through in-depth interviews with the athletes or their guardians, depending on the individual's situation. Two members of the research team (Fahad and Rifa) conducted the interviews, along with a former athlete with a disability who is also the Executive Director of a disability sports club. This individual holds a Master’s degree in Special Education and has 28 years of experience in the field. The data analysis process included several stages:   1. The researchers transcribed the interview and focus group recordings using Sonix (<https://sonix.ai/>), a platform designed for this purpose that supports Arabic and employs artificial intelligence for language and text recognition across contexts. 2. An exploratory content-driven analysis approach was used, which involves open coding and themes derived from the data, applicable to both probabilistic and non-probabilistic samples (Guest et al., 2013). 3. During the qualitative data analysis, the researchers performed triangulation of raw analysis by reviewing the data analysis process with a peer—a colleague in a gifted education PhD program—and a special education expert with 28 years of practical experience, known as peer review (Johnson & Christensen, 2019), to ensure the credibility of the analysis and review the main and sub-themes. 4. Additionally, the researchers presented the final analysis results to an independent researcher for external audit, a method involving external experts to assess the study's quality. The researcher reviewed the primary analysis and the initial review to determine the level of agreement and ensure the quality of identifying the main and sub-themes (Johnson & Christensen, 2019). |
| 3 | In lines 163-167, the authors mention that interview data were collected from 21 respondents on 1 January 2023. The authors state that these interviews add up to 26 hours of documentation. However, due to the relatively large number of 21 respondents, this seems difficult to achieve with only one researcher in a day. It may be necessary for the authors to clarify how interview data were collected, e.g., were the interview data collected on the same day by different researchers? | The mechanisms for conducting the interviews and their chronological sequence are outlined in Table 3 he interviews commenced on October 19, 2022, and concluded on January 9, 2023. Two members of the research team, along with an independent athlete with extensive experience in sports for individuals with disabilities and an executive manager of a disability sports club in the Kingdom, collectively conducted 12 interviews, representing 57% of the interviews. Additionally, a team member and the independent athlete conducted 7 face-to-face interviews, accounting for 33%, while the researcher conducted 2 interviews with female athletes to ensure their willingness to participate in the study, representing approximately 10%. |
| Results | | |
| 1 | In line 206, the authors mention “Identifying the Identity of Twice-Exceptional Elite Athletes: A Mega Mode Approach”, but then go on to say that “This framework categorizes these factors into internal and external components, both of which are integral to motivating individuals toward achieving excellence.” The authors need to clarify whether or not the concept of identity is an external component. | Talent development requires both internal and external factors, which integrate to ultimately foster talent. This principle forms the foundation of the Mega Model of Talent Development (Subotnik et al., 2011). In this study, we explore the internal factors that contribute to the development of talent among elite twice-exceptional athletes. To achieve this, we classified the factors according to the Mega Model into internal components that drive individuals toward excellence. These components include general ability, field-specific skills, psychological and social skills, and experience growth (Olszewski-Kubilius et al., 2022). External components are related to appropriate opportunities and resources in terms of their timing, type, and duration (Preckel et al., 2020). The integration of these components leads to positive talent growth. |
| 2 | In lines 206-239, the authors mention the mega mode approach as the analytical framework for the study. However, the authors did not explain the approach in the article introduction or in the methodology section. It is recommended that the authors include the approach in the literature review. This includes how it has been used in existing studies and how it was used in this study to analyze the findings. As a result, the discussion section will be more able to reflect on and comment on the approach's applicability | A brief explanation of the Mega Model has been provided.  Subotnik, Olszewski-Kubilius, and Worrell (2011) proposed the Mega Model of Talent Development, integrating their extensive experiences and research in the field of talent development. This model draws on a variety of theories, including Bloom's Model of Talent Development (Bloom, 1985), Sternberg's concept of expertise (Sternberg, 1998), Csikszentmihalyi's notion of big and small creativity (Csikszentmihalyi, 1996), and the model of scientific/artistic productivity. Subotnik et al. divided the proposed model into three main stages, as illustrated in Figure (00), and established a set of core principles guiding the model, summarized as follows: (a) General and specific abilities are important and can be developed; (b) Talent domains have different developmental pathways; (c) Opportunities are a key component in talent development; (d) Psychological and social variables are crucial in nurturing talent; (e) Reputation represents the intended outcome of gifted education.    Taken from (Subotnik et al., 2011)    Figure: Mega Talent Model  Taken from (Subotnik et al., 2011)  The model encompasses many influencing factors and integrates previous studies and research in the field of talent development and nurturing. In my personal opinion, based on the earlier presentation of various talent development studies and the historical context of the field, it can be said that Subotnik and colleagues have successfully crafted a synthesis of educational literature in this domain. They have consolidated the key components that educational studies have demonstrated to play a significant role in building and developing individuals' talents across various fields. |
| 3 | The authors mention in lines 225-226 that “Despite the diversity in participants’ disabilities and levels of athletic excellence, our study revealed striking similarities in the experiences of elite athletes”. However, there may be a misunderstanding in the current authors’ generalization of the influencing factors derived from all the respondents. For example, these subjects came from different sports and may be consistent in internal influences. However, they may show a higher degree of variation in external factors. Therefore, a comparative analysis of respondents with varying background conditions should be presented in the findings section. In addition, the research methodology should be categorized according to the different characteristics of respondents | The comparative analysis of external factors has been conducted and is attached in Table 2 of the file. |
| 4 | The authors asked the interviewees to describe their experiences of outstanding athletic performance. From this, the authors extracted the influencing factors and attempt to present the relationships between the factors. Due to the small sample size, the authors will have to clarify the relationship of the factors between each other carefully. For example, does “Perseverance” also affect “Desire for Self-assertion”? Therefore, it is suggested that the authors focus on the induction of the influencing factors rather than the relationship between the factors. | The experiences reported by the participants revealed that there is an interactive stimulation among internal components, as well as among environmental components. Additionally, there is a positive and motivating interaction between these internal and environmental components, where each factor stimulates the emergence and growth of the other, depending on the age and developmental stage of the talent.  The model describes the role of deliberate practice in improving performance and accelerating towards excellence (Preckel et al., 2020). While the initial inclination towards the sports path was similar among participants, the varying capacities represented in the interaction of deliberate practice methods were different. This led to variations in skill development and achievement levels.  For instance, the adolescent phase, which was the starting point for most participants' sports careers, often begins with the development of functions across various sports domains (Olszewski-Kubilius et al., 2022). This phase was stimulating for certain emotional traits such as a love for adventure and challenge, and a desire for self-achievement (Eccles, 2009). However, differences in parental educational and economic levels, as well as the opportunity for discovery related to geographical location, affected the emergence of emotional traits in motivating the development of sports talent. Athletes who had the right opportunities and resources interacted optimally, achieving high levels of performance, setting records in their sports, and winning numerous gold medals globally. In contrast, those without similar resources or opportunities for good sports upbringing were significantly lower in skill and achievement levels. This interaction was not the sole cause of the developmental variance; the timing of these resources and their interaction was another influential factor in good upbringing. We found that a suitable geographical location with available services and awareness, combined with good economic and educational levels of the parents during the early stages of twice-exceptional individuals (before talent emergence), helped them develop their sports abilities better and achieve higher levels of accomplishment compared to those who did not have such opportunities early in life. The timing of obtaining the right opportunities, according to the Mega Model, is a crucial factor for sustaining development, as each domain has specific temporal needs for effective upbringing (Subotnik et al., 2011). Mere availability of resources, without systematic organization of their type, quantity, and timing, would hinder the control over their interaction and positive impact on talent development, skill growth, and achievement level.  The model also indicates a gradual development of abilities until they become competencies, followed by reaching expertise and then excellence in the field (Subotnik et al., 2017a). We found that transitioning from the ability level to competency was easier for elite athletes than transitioning from competency to expertise. This required a higher level of internal components to continue improving performance and overcoming competitors, while environmental factors like educational and economic levels, and geographical location had less impact. The reason for this lies in the strong internal drive towards achievement and its direct connection to self-fulfillment, which has been termed as "falling in love" (Subotnik et al., 2010). This drive acted as a significant internal motivator for mastering performance and improving psychological, social, and life skills (Celenk, 2021). Research results have highlighted the importance of these internal emotional components, such as passion, desire for satisfaction, and self-achievement, and their role in enhancing athletic performance when combined with deliberate practice (Verner-Filion et al., 2017). The development of this motivation at this stage was crucial as it enabled individuals to continue advancing and sustaining progress. It was found that passion and intense emotions, sometimes described as obsession, affect athletes in various ways. The immediate effect is through the sense of achievement and vitality in moments of victory, while the implicit effect is long-term, as emotions indirectly motivate athletes to set personal goals and adopt individual methodologies leading to mastery (Li, 2010).  Elite athletes at this stage also need psychological and social skills to handle difficulties, self-promotion, confront challenges, and surpass competitors. These skills may arise from deep engagement in the field and achieving expertise in sports practice, as reported by elite athletes in the Paralympic Games, where more experienced individuals developed psychological and social skills like leadership and communication abilities (Morais Freire et al., 2021). Elite athletes also need to learn specific secrets of professionalism and performance arts, hence requiring highly skilled experts and coaches to perform automatically and handle difficult challenges successfully (López-Flores et al., 2021). This was confirmed by participants discussing intensive training camps and how professional coaches contributed to their accelerated transition to excellence and expertise in their field.  We observed the variation in the importance of internal and external components across different developmental stages. We noted how the presence of a component at the right time to complete the developmental path and achieve exceptional talent development is crucial. Conversely, the delay in the presence of such components can hinder development. The issue of timing was fundamental to the Mega Model, and this timing, along with the quantity, type, and nature of components, will not be identical across all domains; each domain has its specific temporal needs. |
| Discussion | | |
| 1 | The authors describe, in lines 415-418, “Given the diverse experiences of the participants within the twice-exceptional category, we conducted comparative analyses to benchmark their actions’ efficacy and their influence on developmental trajectories under varying circumstances, contrasting ideal conditions with those lacking such support”. However, the results of the comparative analyses were not found in the study findings. This section also responds to the question in the findings section whether comparative analyses of data from respondents with different background conditions should be presented first. It is therefore suggested that the authors should revise their discussion to be more in line with the study findings. | The comparative analysis of the external factors has been conducted and is attached in the file at Table 2. |
| 2 | The current discussion by the authors takes place at the same time when the research findings are in dialogue with previous literature or theoretical applications, e.g. lines 425-444. Apart from the fact that readers may get lost easier in a large amount of text, the discussion of the research findings and previous related studies seems incomplete. Therefore, it is suggested that the authors move the discussion of the findings of internal and external factors in the Results section, such as the similar content in lines 367-375, to the Discussion section for a more comprehensive discussion, as well as discussing them according to the themes, which may make it easier for readers to understand the themes that the authors would like to focus on. | Done from line 366 to 368 |
| Problems with Article Format | | |
| 1 | What is the keyword 6.? | The Mega Model of Talent Development is a framework proposed by Subotnik et al. (2011) to explain how talent develops through internal and external factors:  Internal Factors:  General Abilities: Basic mental and physical capabilities.  Specialized Abilities: Skills and knowledge in a specific area.  Psychological and Social Skills: Motivation, self-confidence, and coping with stress.  Experience Growth: Accumulating and refining experience through practice.  External Factors:  Opportunities and Resources: Available resources, social support, and training opportunities.  Environment: Quality of education, guidance, and available resources.  Interaction:  Between Internal and External Factors: How they work together to support development.  Among Internal Components: How different internal factors influence each other.  Developmental Stages:  Progression: From ability to competence, then to expertise, and finally to excellence.  The model is used to design training programs, evaluate performance, and guide research on talent development. It emphasizes the importance of deliberate practice and available opportunities for achieving success. |
| 2 | Missing spelling, e.g., line 68 "familial"; line 376 "his factor"; Table 2 title “Environmental Facto”. | Done |
| 3 | Names and dates of people appear in internal citations, e.g., lines 211-214 and 215-217 | Done |
| 4 | Why do the external components in line 215 appear in bold, but the internal components in line 211 do not? | Done |
| 5 | Lines 248-249 repeat the phrase "with unique abilities and challenges” | The repetition was deleted |
| 6 | The font size is inconsistent in many places throughout the text, e.g., in the introduction and lines 152-153, Table 1, etc. 7. The age group in Table 1 contradicts the font size. | Done |
| 7 | In Table 1, there should be no brackets around the age group | Brackets was deleted |
| 8 | In Table 1, there should be no need to add punctuation marks for the sports items above Shot put. | Done |
| 9 | Figure 2 is quite blurry and the font size is small. | Done |
| 10 | The spacing between paragraphs is inconsistent. | Done |
| 11 | References section lacks a more carefully compiled list, e.g. lines 527, 534, 554. | Done |
| Reviewer #2 |  |  |
| 1 | : This manuscript examines the factors affecting talent development and athletic performance among Saudi Arabia's Twice-Exceptional Athletes, focusing on both internal psychological factors and external environmental influences. The study is underpinned by qualitative interviews and explores how these factors contribute to success at regional and international Paralympic competitions across various sports and regions within the Kingdom. The selection of the sample and variables could be enhanced by including education level and profession as indicators of financial support and self-funding. This addition is particularly relevant given the disparities in financial backing and media coverage between Paralympic sports and other athletic disciplines. Moreover, the paper would benefit from a deeper contextualization within the current sports policy framework in Saudi Arabia, especially concerning disability and Paralympic sports. This should encompass aspects like financial support, dual careers, and access to facilities. The discussion on regional discrepancies in success factors for the studied population is somewhat generic. A more detailed examination of specific cities that provide optimal conditions for these athletes would add value. Furthermore, the gender dimension warrants more comprehensive analysis and discussion. | The economic and occupational levels, as well as geographic and gender variables of the participants, were specified in Table 1 (Participant Data) and their impact on performance levels was compared in Table 2.  Additionally, a brief section was added regarding the Paralympic sports policy in Saudi Arabia and the impact of recent changes in the field. |
|  | There are also several stylistic concerns that require the authors' attention: Ensure font consistency throughout the document. For example, the font in Table 2 differs from the rest of the paper. Standardize table formatting to enhance readability, applying the style used in Table 1 to all subsequent tables. Correct typographical errors, such as the one on line 367; it should read "this factor" instead of "his factor | Done |
| 6 | PLOS authors have the option to publish the peer review history of their article (what does this mean?). If published, this will include your full peer review and any attached files. If you choose “no”, your identity will remain anonymous but your review may still be made public. Do you want your identity to be public for this peer review? For information about this choice, including consent withdrawal, please see our Privacy Policy.  Reviewer #1: No  Reviewer #2: No |  |
