# Supplementary material for: The internal and external factors influencing talent development of athletic talent among Saudi Arabia’s twice exceptional elite athletes: A comprehensive study
Source: PLoS One. 2024 Dec 27;19(12):e0304049. doi: 10.1371/journal.pone.0304049 (PMC11676560; doi:10.1371/journal.pone.0304049)
Supplement: Attachment — Submitted filename: Review Comments.docx [file pone.0304049.s002.docx]

Greetings, authors,

Thank you for modifying and responding to our suggestions. Overall, the quality of the current study has improved. However, there are still a few points to consider and clarify. Here are some suggestions to consider before submitting the article to a journal.

**Materials and Methods**

1. Although the authors responded, "All requirements have been added to Table (1)", when reviewing Table 1, we cannot identify the respondent code, the respondent's disability classification, and the sport he/she plays. As an aside, the document currently has two Tables 1.
2. The author responds by saying, "The following passage has been added to clarify the validity of the interviews and qualitative data analysis.". It is in line with our recommendation. A review of the authors' manuscript, however, does not indicate that it has been added.
3. Also, although the authors responded to our suggestions, they did not include this information in their section on data collection.

**Results**

1. In line 206, the authors mention “Identifying the Identity of Twice-Exceptional Elite Athletes: A Mega Mode Approach”, but then go on to say that “This framework categorizes these factors into internal and external components, both of which are integral to motivating individuals toward achieving excellence”. In psychology, identity refers to traits, beliefs, personalities, appearances, and/or expressions that define a person or group. Therefore, it should be an internal component. In this context, it might be more appropriate for the author to use the term "influencing factors" rather than "identity."
2. In table 2, there are still no changes that would respond to our previous suggestions regarding the presentation of "The comparative analysis of external factors".
3. Only one change has been made to the separation of the findings from the discussion of previous studies. It is necessary to make many other adjustments, such as in lines 264-266.

**Problems with Article Format**

Throughout the article, there are several formal errors that need to be corrected by the author. The following is just a list of cases, and authors are encouraged to check each one throughout the text.

1. There is no space before [13] on line 52.
2. There is a need to clarify line 91.
3. There is a difference in font size between line 203 "Results" and other headings of the same level.
4. There is a need to adjust the distance between the lines on lines 318-319.
5. There is an inconsistency in the font size for Line 320.
6. The following lines are garbled: 523, 652, 717, 726.
