# Supplementary material for: The internal and external factors influencing talent development of athletic talent among Saudi Arabia’s twice exceptional elite athletes: A comprehensive study
Source: PLoS One. 2024 Dec 27;19(12):e0304049. doi: 10.1371/journal.pone.0304049 (PMC11676560; doi:10.1371/journal.pone.0304049)
Supplement: Attachment — Submitted filename: Response to Reviewers.docx [file pone.0304049.s003.docx]

Dear Dr. Tien-Chin Tan,

Thank you once again for your thoughtful and constructive feedback on our manuscript. We sincerely appreciate the time and effort you have dedicated to reviewing our work.

In response to your comments from the second round of reviews, we have made significant revisions to the manuscript to address your concerns and suggestions. Your insights have been invaluable in guiding us to improve the clarity, coherence, and overall quality of the research.

In this table you will find all the required responses and modifications.

Respond to all comments received via email

| No. | Comments | Response |
| --- | --- | --- |
| Materials and Methods | | |
| 1 | Although the authors responded, "All requirements have been added to Table (1)", when reviewing Table 1, we cannot identify the respondent code, the respondent's disability classification, and the sport he/she plays. As an aside, the document currently has two Tables 1. | A new table was added with the number Table 1, which describes the participants according to the type of sport, type of disability, age, and timing of disability. Each participant was mentioned by his code, and the title of the table was Participant Distribution by Sport Type, Disability Type, Age, and Timing of Disability. |
| 2 | The author responds by saying, "The following passage has been added to clarify the validity of the interviews and qualitative data analysis.". It is in line with our recommendation. A review of the authors' manuscript, however, does not indicate that it has been added. | The response to what the reviewers requested has been referred to and added to the manuscript. |
| 3 | 3. Also, although the authors responded to our suggestions, they did not include this information in their section on data collection. | Don,  we added  The following passage has been added to clarify the credibility of the interviews and qualitative data analysis:  Data from the participants were collected using the study tool through in-depth interviews with the athletes or their guardians, depending on the individual's situation. Two members of the research team (Fahad and Rifa) conducted the interviews, along with a former athlete with a disability who is also the Executive Director of a disability sports club. This individual holds a Master’s degree in Special Education and has 28 years of experience in the field. The data analysis process included several stages:  1. The researchers transcribed the interview and focus group recordings using Sonix (https://sonix.ai/), a platform designed for this purpose that supports Arabic and employs artificial intelligence for language and text recognition across contexts.  2. An exploratory content-driven analysis approach was used, which involves open coding and themes derived from the data, applicable to both probabilistic and non-probabilistic samples[49].  3. During the qualitative data analysis, the researchers performed triangulation of raw analysis by reviewing the data analysis process with a peer—a colleague in a gifted education PhD program—and a special education expert with 28 years of practical experience, known as peer review (Johnson & Christensen, 2019), to ensure the credibility of the analysis and review the main and sub-themes [48].  4. Additionally, the researchers presented the final analysis results to an independent researcher for external audit, a method involving external experts to assess the study's quality. The researcher reviewed the primary analysis and the initial review to determine the level of agreement and ensure the quality of identifying the main and sub-themes[48]. |
| **Results** | | |
| 1 | In line 206, the authors mention “Identifying the Identity of Twice-Exceptional Elite Athletes: A Mega Mode Approach”, but then go on to say that “This framework categorizes these factors into internal and external components, both of which are integral to motivating individuals toward achieving excellence”. In psychology, identity refers to traits, beliefs, personalities, appearances, and/or expressions that define a person or group. Therefore, it should be an internal component. In this context, it might be more appropriate for the author to use the term "influencing factors" rather than "identity." | The term has been changed and modified in the manuscript on line 252 to influencing factors//// instead of Identity. |
| 2 | In table 2, there are still no changes that would respond to our previous suggestions regarding the presentation of "The comparative analysis of external factors". | We have made significant changes to address suggestions for a “comparative analysis of external factors.” A comprehensive comparison of various environmental factors has been included, including academic and economic levels, geographic location, timing of injury, talent discovery, and intensive training. The impact of each of these factors, both positive and negative, on athletes’ performance is illustrated, reflecting the impact of the surrounding environment on their success in sport. With this amendment, we hope that the information presented in Table 4 will be clearer and more comprehensive, meeting the reviewer’s expectations for an effective comparative analysis. |
| 3 | Only one change has been made to the separation of the findings from the discussion of previous studies. It is necessary to make many other adjustments, such as in lines 264-266. | All modifications have been made and added. |
| Problems with Article Format | | |
|  | There is no space before [13] on line 52. | Done |
|  | There is a need to clarify line 91. | Done |
|  | There is a difference in font size between line 203 "Results" and other headings of the same level. | Done |
|  | There is a need to adjust the distance between the lines on lines 318-319. | Done |
|  | There is an inconsistency in the font size for Line 320. | Done |
|  | The following lines are garbled: 523, 652, 717, 726. | Done |
| Editor Comment | | |
| 1 | We cannot proceed unless you comply with the following requirements. Please provide response in your cover letter. | Done |
| 2 | We note that several of your files are duplicated on your submission. Please remove any unnecessary or old files from your revision, and make sure that only those relevant to the current version of the manuscript are included. | Done |
| 3 | Thank you for stating the following financial disclosure: [In The authors extend their appreciation to the King Salman center For Disability Research for funding this work through Research Group no KSRG-2022-090. ].  Please state what role the funders took in the study. If the funders had no role, please state: "The funders had no role in study design, data collection and analysis, decision to publish, or preparation of the manuscript." | "The authors extend their appreciation to the King Salman center For Disability Research for funding this work through Research Group no KSRG-2022-090. The funders had no role in study design, data collection and analysis, decision to publish, or preparation of the manuscript." |
