# Supplementary material for: The internal and external factors influencing talent development of athletic talent among Saudi Arabia’s twice exceptional elite athletes: A comprehensive study
Source: PLoS One. 2024 Dec 27;19(12):e0304049. doi: 10.1371/journal.pone.0304049 (PMC11676560; doi:10.1371/journal.pone.0304049)
Supplement: Attachment — Submitted filename: Response to Reviewers.docx [file pone.0304049.s004.docx]

Dear Dr. Tien-Chin Tan,

Thank you once again for your thoughtful and constructive feedback on our manuscript. We sincerely appreciate the time and effort you have dedicated to reviewing our work.

In response to your comments from the third round of reviews, we have made significant revisions to the manuscript to address your concerns and suggestions. Your insights have been invaluable in guiding us to improve the clarity, coherence, and overall quality of the research.

In the attached table, you will find detailed responses to all comments received via email, along with the corresponding modifications made to the manuscript.

Additionally, we would like to kindly inform you that our research team is hopeful for an expedited review process. This will enable us to submit the manuscript to the funding agency before the previously mentioned deadline of 30 November, ensuring we can present it to the King Salman Center for Disability Research in a timely manner.

Thank you for your continued support and understanding.

| No. | Comments | Response |
| --- | --- | --- |
| **Journal Requirements:** | | |
| 1 | Please review your reference list to ensure that it is complete and correct. | The reference list has been thoroughly reviewed to ensure it is complete and accurate. We also use the EndNote application for writing and managing references. |
| 2 | If you have cited papers that have been retracted, please include the rationale for doing so in the manuscript text, or remove these references and replace them with relevant current references. | Thank you for your note regarding retracted papers. I would like to confirm that I have thoroughly reviewed the references cited in my manuscript, and none of them include retracted papers. |
| 3 | Any changes to the reference list should be mentioned in the rebuttal letter that accompanies your revised manuscript. | References 77 to 83 have been updated in the revised reference list.  References 78 to 77 have been updated in the revised reference list.  References 81 to 78 have been updated in the revised reference list.  References 82 to 79 have been updated in the revised reference list.  References 80 to 85 have been updated in the revised reference list.  References 83 to 80 have been updated in the revised reference list.  References 84 to 81 have been updated in the revised reference list.  EndNote automatically updates the reference list in the manuscript body and reference list. |
| 4 | If you need to cite a retracted article, indicate the article’s retracted status in the References list and also include a citation and full reference for the retraction notice. | I confirm that my manuscript does not include citations from retracted articles. All references have been carefully reviewed to ensure their validity and relevance. |
| **Additional Editor Comments:** | | |
| 1 | Thanks for revising the original manuscript. In the revised manuscript, there have been improvements. Although enhancements have been made, reviewers remain concerned about certain issues. | Thank you for your positive feedback on the revisions made to the manuscript. We are glad that the improvements have been noted, and we appreciate your acknowledgment of the changes. We will continue to work on refining the manuscript further to address any remaining concerns. |
| 2 | Please make an adjustment or provide clarification to clarify the reviewers' concerns. The manuscript can be accepted after a few minor revisions. Looking forward to receiving the further revised version. | Thank you for your feedback and for recognizing the improvements made in the revised manuscript. We appreciate the reviewers' insightful comments and understand that some concerns remain. We are currently working on addressing these points by making the necessary adjustments and providing clarifications to ensure the manuscript fully meets the required standards. We will carefully review the reviewers' concerns and make the appropriate revisions to resolve any remaining issues. Thank you again for your valuable feedback, and we hope that these amendments will be comprehensive, provide all clarifications, and remove all concerns. |
| **Comments to the Author  Materials and methods** | | |
| 1 | The authors have added Table 1 to the revised version of the article to show the Participant Distribution. This allows readers to understand the respondents' attributes. However, in general, the authors will point out the location of the table and explain the meaning of the table in the text. | We appreciate your valuable comment. In response, we have included a clear reference to Table 1 in the appropriate section of the manuscript, immediately following the table. Additionally, we have provided a detailed explanation of its content within the text to help readers better understand the participant distribution and the significance of the data presented in the table. The explanation is as follows: Table 1 highlights the distribution of volunteer participants in the study based on sport type, disability type, age, and disability onset. The key findings from Table 1 can be summarized as follows:  Participant Diversity (Age and Disability Type):  Table 1 reveals significant diversity in age groups, with participants ranging from 19 years old (the youngest) to 56 years old (the oldest).  There is also notable variation in disability types, including physical impairments such as paraplegia and limb amputations, as well as intellectual disabilities and autism spectrum disorders.  Disability Onset and Causes:  Disabilities are categorized into those present since birth or early childhood (e.g., polio or oxygen deprivation during birth) and those acquired later due to accidents or medical errors (e.g., injuries caused by accidents or vaccination complications).  The timing of disability onset reflects its psychological and social impact, with some participants acquiring their disabilities during critical life stages, such as adolescence or adulthood.  Sport Type:  Participants engage in a wide range of sports that require diverse physical abilities:  Individual sports: such as powerlifting, track and field (e.g., shot put, wheelchair racing).  Team sports: such as wheelchair basketball.  Para-specific competitive sports: including Boccia and classification-based sports (e.g., T and F categories).  This diversity demonstrates the opportunities available for individuals with disabilities to participate in various sports, taking into account the specific classifications associated with each disability type. |
| **Results** | | |
| 1 | Lines 378-403, both paragraphs are identical. | Thank you for pointing that out. In response, we have removed the duplicated content between lines 378-403 to ensure clarity and avoid repetition. |
| **Discussion** | | |
| 1 | lines 542-544, there is a problem with the subparagraphs. 2. | Thank you for pointing out the problem in lines 542 to 544 regarding the subparagraphs. I have reviewed the content and made the necessary adjustments to ensure clarity and proper formatting. This was done by modifying and arranging the paragraphs of the entire discussion and placing headings so that the reader can easily browse the discussion |
| 2 | Suggest that the author use a topic-based presentation in the discussion section to make it easier for the reader to understand what the author is trying to discuss. | Numerous studies in the literature have affirmed the potential for intentional development across various domains [76]. However, a pertinent question arises: What were the key factors responsible for nurturing sports talent among elite, twice-exceptional athletes?.  To elucidate these capacities and comprehend their operational mechanisms, we scrutinized the experiences of individuals who had attained the benchmarks indicative of talent. Accordingly, we investigated both internal factors and environmental factors, discerning how they catalyzed talent development. Given the diverse experiences of the participants within the twice-exceptional category, we conducted comparative analyses to benchmark their actions' efficacy and their influence on developmental trajectories under varying circumstances, contrasting ideal conditions with those lacking such support.  The participants' experiences unveiled a synergistic interplay among internal factors and other components, alongside interactions with environmental factors, both individually and collectively. Furthermore, a positive and catalytic interaction was observed between internal and environmental factors. Each factor stimulated the emergence and evolution of the other, contingent upon the individual's age and developmental stage within the talent trajectory.  The role of internal factors in developing sports performance:  The transition into the realm of sports followed a similar trajectory for participants, yet the nuanced interplay of various capabilities, representing the intended practice method, differed significantly. This variance influenced the breadth and depth of skill development achievement. For many participants, adolescence marked the on-set of their sports journey, coinciding with the commencement of diverse sporting pursuits[51]. These endeavors often catalyzed emotional qualities such as a love for challenge and self-assertion [76].  Motivation serves as an internal engine for mastering performance and improving psychological, social, and life skills [77]. Previous research emphasizes the significance of internal emotional components such as passion, satisfaction, desire, and self-assertion in enhancing athletic performance within structured practice environments[63]. Passion and strong emotions, sometimes referred to as "hallucinations," have varied effects on athletes. They not only contribute to feelings of achievement and vitality during moments of victory but also catalyze athletes to set objectives and adopt individual methodologies, ultimately leading to excellence[64].  Studies centered on the hypotheses underlying the model and the requirements of the twice-exceptional population emphasized the importance of leveraging strengths, addressing weaknesses, implementing credible interventions, and tailoring approaches to individual needs[78-81]. These efforts were aimed at fostering supportive internal psychological traits, such as passion, perseverance, and assertiveness, which were crucial for talent development.  Notably, many individuals struggled to accept their new circumstances, which hindered their participation in sports activities. This finding is consistent with research by Boyce and Wood (2011), which suggests that adapting to a disability is easier when it occurs earlier in life. In contrast, those who adapted successfully achieved remarkable accomplishments and competed at various levels, balancing their athletic pursuits with other aspects of life.[73, 82]. Moreover, Gignac et al. (2000) observed that individuals with chronic disabilities often develop effective coping mechanisms, further emphasizing the importance of psychological resilience in overcoming challenges[73]. These insights highlight the importance of early adaptation to disability and the role of active compensatory strategies in achieving success in sports and maintaining a balanced life.  Motivation surged when favorable environmental factors were aligned with internal traits, enabling disabled individuals to achieve remarkable performance commensurate with their age and athletic trajectory. These internal components served as catalysts for overcoming challenges and sustaining motivation and growth.  Our findings indicate that elite athletes navigate this transition from capability to competence more easily than from competence to experience. Internal components play a crucial role in this process, enabling athletes to enhance their performance and surpass competitors. While environmental factors like academic and economic levels, as well as geographical location, have less influence, internal motivation for achievement and self- assertion emerges as key drivers, often described as falling in love [19].  The role of external factors in developing sports performance:  Disparities in parental education and economic status, along with opportunities for talent discovery linked to geographical location, significantly influenced the development of emotional qualities essential for nurturing sports talent. Champions who benefited from supportive environments and optimal interactions achieved exceptional performance levels, earning numerous international gold medals and setting records. In contrast, those lacking such nurturing opportunities did not attain comparable levels of skill and accomplishment. While interaction dynamics played a crucial role, the presence of abilities and the timing of their development also impacted outcomes. Geographical suitability, coupled with parental support and educational advantages in early life, facilitated the athletic development of twice-exceptional individuals, enhancing their chances of achieving high levels of success. According to the "Mega Model," timely opportunities were critical for sustained growth, as each domain required specific timing for optimal development [51]. It is evident that capabilities, when coupled with a systematic arrangement of quality, size, and timing, facilitate a controlled interaction process with a positive impact on skill development and achievement levels.  The model suggests that capabilities undergo a developmental transition toward competence, leading individuals to become experienced and eventually distinguished in their field[83].  This stage demanded that elite athletes possess psychological and social skills essential for navigating challenges, asserting themselves, confronting obstacles, and outperforming competitors. These skills are often cultivated through immersion in the field and gaining experience through sports practices, as evidenced by elite athletes' experiences in the Olympic Games for the disabled. Research suggests that individuals with more experience tend to develop psychological and social skills such as leadership and communication[84]. Additionally, elite athletes needed to master the intricacies of professionalism and performance arts, underscoring the importance of having expert coaches with a high level of professionalism to facilitate seamless performance and successful navigation of challenges [85]. Participants highlighted the significance of boot camps and the role of professional trainers in accelerating their transition towards excellence and expertise in the field.  We observed the divergence between internal and external factors across various stages of development and noted how the timing of these components influenced talent development trajectories, particularly in the case of twice-exceptional individuals. Timing emerged as a fundamental aspect of the Mega Model theory, wherein the presence of components at opportune moments significantly impacted talent development outcomes. Additionally, each domain had unique temporal needs, highlighting the importance of considering timing in talent development strategies. |
| **Problems with the Article Format** | | |
| 1 | On line 119, citation formatting should be standardized. In every section of the article, fonts should be used. | Done As noted by Moser and Korstjens, face-to-face in-depth interviews are the most propitious for phenomenological research methodology. Similarly, Creswell and Poth emphasized this method [40, 45]. |
| 2 | It would be helpful if you standardized the font and font size throughout the article. | Done |
| 3 | On lines 538 and 546, please delete the extra parentheses. | Done |
|  |  |  |
|  |  |  |
|  |  |  |
